# IFN-γ could induce ferroptosis in keloid fibroblasts by inhibiting the expression of serpine2

**DOI:** 10.1038/s41420-025-02401-3

**Published:** 2025-05-05

**Authors:** Jingyan Huang, Shun Yu, Jing Luo, Xusong Luo, Jun Yang, Xiuxia Wang

**Affiliations:** 1 Department of Plastic and Reconstructive Surgery, Shanghai Ninth People’s Hospital, Shanghai Jiaotong University School of Medicine, Shanghai, China; 2 The Affiliated Hospital of Jiangnan University, Wuxi, Jiangsu China

**Keywords:** Apoptosis, Skin diseases

## Abstract

Keloids are common pathological scars resulting from previous trauma or inflammation. Interferon-gamma (IFN-γ) has shown significant therapeutic effects when used alone or in combination with other agents. While IFN-γ has been found to regulate ferroptosis in tumor cells, its ability to regulate ferroptosis in keloid fibroblasts (KFs) is unclear. Here, we have demonstrated a direct causal relationship between IFN-γ levels and ferroptosis in KFs. To explore the intrinsic mechanism, we performed genome-wide RNA and proteomics sequencing and found that serpine2 was the most significantly downregulated gene in KFs after exogenous overexpression of IFN-γ. Serpine2, which belongs to a family of serine protease inhibitors, has been shown to play an important role in fibrotic diseases. Therefore, we hypothesized that serpine2 is a downstream gene in the regulation of ferroptosis in KFs by IFN-γ. Our results showed that serpine2 overexpression promotes collagen synthesis, which in turn promotes the proliferation, migration, and invasive functions of KFs. We further demonstrated that serpine2 overexpression promoted system Xc^−^ transporter expression, cystine uptake, and glutathione synthesis, enhanced GPX4 activity; and inhibited reactive oxygen species generation. This resulted in a reduction in intracellular lipid peroxidation and the levels of its metabolite malondialdehyde, as well as inhibited ferroptosis in KFs. IFN-γ reversed these effects of serpine2 overexpression. These results were largely confirmed in in vivo keloid models too. These findings imply that IFN-γ not only directly induces ferroptosis in KFs but also enhances their sensitivity to ferroptosis by inhibiting the synthesis of SLC7A11 and SLC3A2 through downregulation of serpine2. In summary, we suggest that the serpine2-system Xc^−^ axis is a promising therapeutic target for the treatment of keloids.

## Introduction

Keloids are common pathological scars that typically occur after inflammation or trauma and are particularly prevalent in individuals with darker skin, affecting 4–16% of individuals [1]. Beyond esthetic concerns, keloids can lead to functional issues such as contractures and cause symptoms such as itching, which can significantly impact the quality of life and physical and mental health [2]. Keloids are treated with silicone gel sheets, compression therapy, radiotherapy, and surgery, but each method is associated with limitations in practice [3]. While surgery can address joint contractures, it does not effectively reduce itching and has less than satisfactory cosmetic outcomes. Moreover, post-surgery recurrence rates are as high as 45–100% [4]. The other therapies are hampered by low efficacy, hyperpigmentation, and high recurrence rates [3].

Another treatment agent for keloids is interferon-gamma (IFN-γ), a type II interferon that plays a crucial role in the immune system through its antiviral, antiproliferative, and immunomodulatory effects [5]. IFN-γ has demonstrated significant therapeutic effects on keloids and was included in the international clinical guidelines for scar management as early as 2002 [6]. IFN-γ can downregulate the expression of collagen types I and III in keloid tissue, counteract interleukin-6 overexpression, antagonize transforming growth factor-beta, and inhibit keloid fibroblast (KF) proliferation. These mechanisms are crucial for keloid treatment because they effectively reduce keloid size and texture while alleviating symptoms [7]. Additionally, IFN-γ has also shown promise for keloids when used in combination with tretinoin, 5-fluorouracil, and bleomycin [7, 8]. Imiquimod 5%, a topical immune response modifier, was found to promote collagen breakdown by stimulating the production of IFN-γ [9]. Further research into IFN-γ mechanisms could help identify strategies to enhance its therapeutic outcomes.

Ferroptosis, a type of regulated cell death, results from excess iron, increased reactive oxygen species (ROS) levels, and an imbalance in unsaturated fatty acid concentrations [10]. Ferroptosis modulation has been reported to be beneficial in the treatment of fibrotic diseases, drug-resistant cancers, and lipid peroxidation-associated degenerative conditions [11–13]. Moreover, modulation of macrophage ferroptosis can reduce dermal fibrosis in animal models of systemic sclerosis [14]. A strong connection between IFN-γ and ferroptosis has recently emerged. IFN-γ secreted by CD8+ T cells, in conjunction with arachidonic acid, was found to induce ferroptosis in tumor cells by targeting the Acyl-CoA synthetase long-chain family member 4 (ACSL4) promoter [15]. Additionally, IFN-γ was found to induce ferroptosis by inhibiting solute carrier family 3, member 2 (SLC3A2), and Solute Carrier Family 7 Member 11 (SLC7A11), reducing cystine uptake in tumor cells and suppressing the glutathione peroxidase 4 (GPX4) signaling pathway [10]. Keloids were found to contain a higher number of IFN-γ-secreting CD8+ T cells than healthy skin tissue [16]. However, it is unclear whether ferroptosis also plays a role in the effects of IFN-γ on KFs. In this study, we aim to fill in the research gap described above by investigating whether IFN-γ alleviated keloids by inducing ferroptosis in KFs and the underlying mechanisms.

## Results

### IFN-γ could inhibit the biological function of KFs and promote its apoptosis

**Fig. 1:**
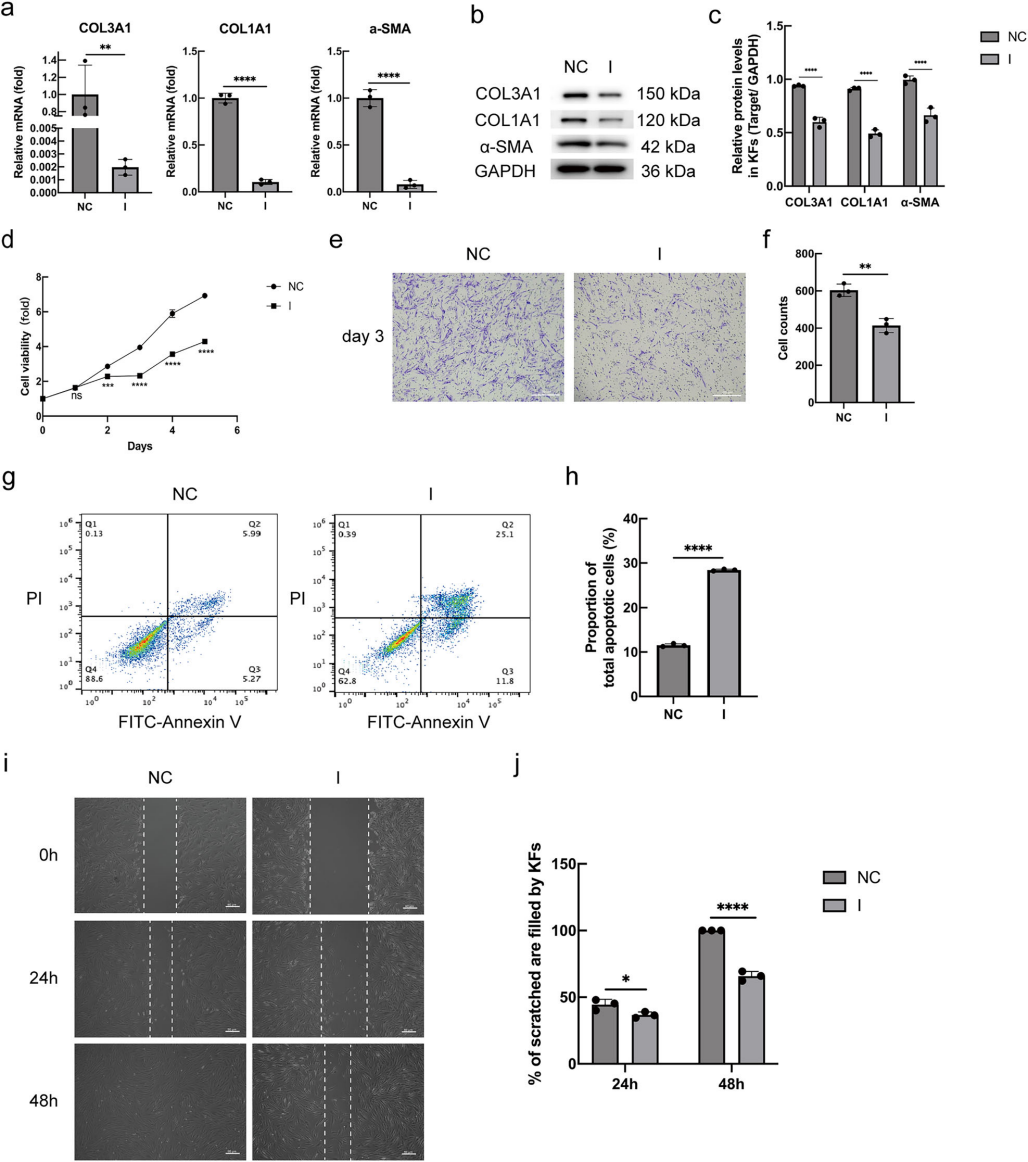
IFN-γ inhibits the proliferation, migration, and invasive ability of KFs. **a**–**c** The gene and protein expression of KFs after being treated with IFN-γ for 24 h. **d** Cell viability of KFs was determined by the CCK8 assay. **e**, **f** Cells were seeded into Transwell chambers, and after 3 days, the number of cells that crossed the filter membrane to the lower layer was counted and tallied. **g**, **h** After incubation with Annexin V-FITC and PI of plasmid-transfected KFs, the flow cytometry was used to calculate the percentage of apoptotic cells in each group. **i**, j Wound healing modeling images were taken at 24 and 48 h after the wound was created. The experiments were conducted in triplicate, and the findings were quantified using the FIJI software. (*n* = 3) (ns not significant; *P < 0.05, **P < 0.01, ***P < 0.001, ****P < 0.0001).

To assess whether IFN-γ could inhibit cell function in primary KFs through an endogenous mechanism, KFs were transfected with an empty control plasmid (NC group) and an IFN-γ overexpression plasmid (I group) by incubation for 24 h. (ELISA kit assay after transfection of IFN-γ overexpression plasmid is shown in Supplementary Fig. 1). The gene and protein levels of collagen types I and III (COL1A1 and COL3A1) in the matrix components of the I group were significantly lower than those of the NC group (Fig. 1a–c). Additionally, IFN-γ induced reduced expression of the α-smooth muscle actin (α-SMA) gene and protein (Fig. 1a–c). Further examination of the effect of IFN-γ on KF function showed that IFN-γ could inhibit the proliferation (Fig. 1d), migration (Fig. 1i, j), and invasive properties (Fig. 1e, f) of KFs and promote their apoptosis (Fig. 1g, h). Thus, IFN-γ appears to have a significant inhibitory effect on KFs’ functions.

### IFN-γ induces ferroptosis in KFs

**Fig. 2:**
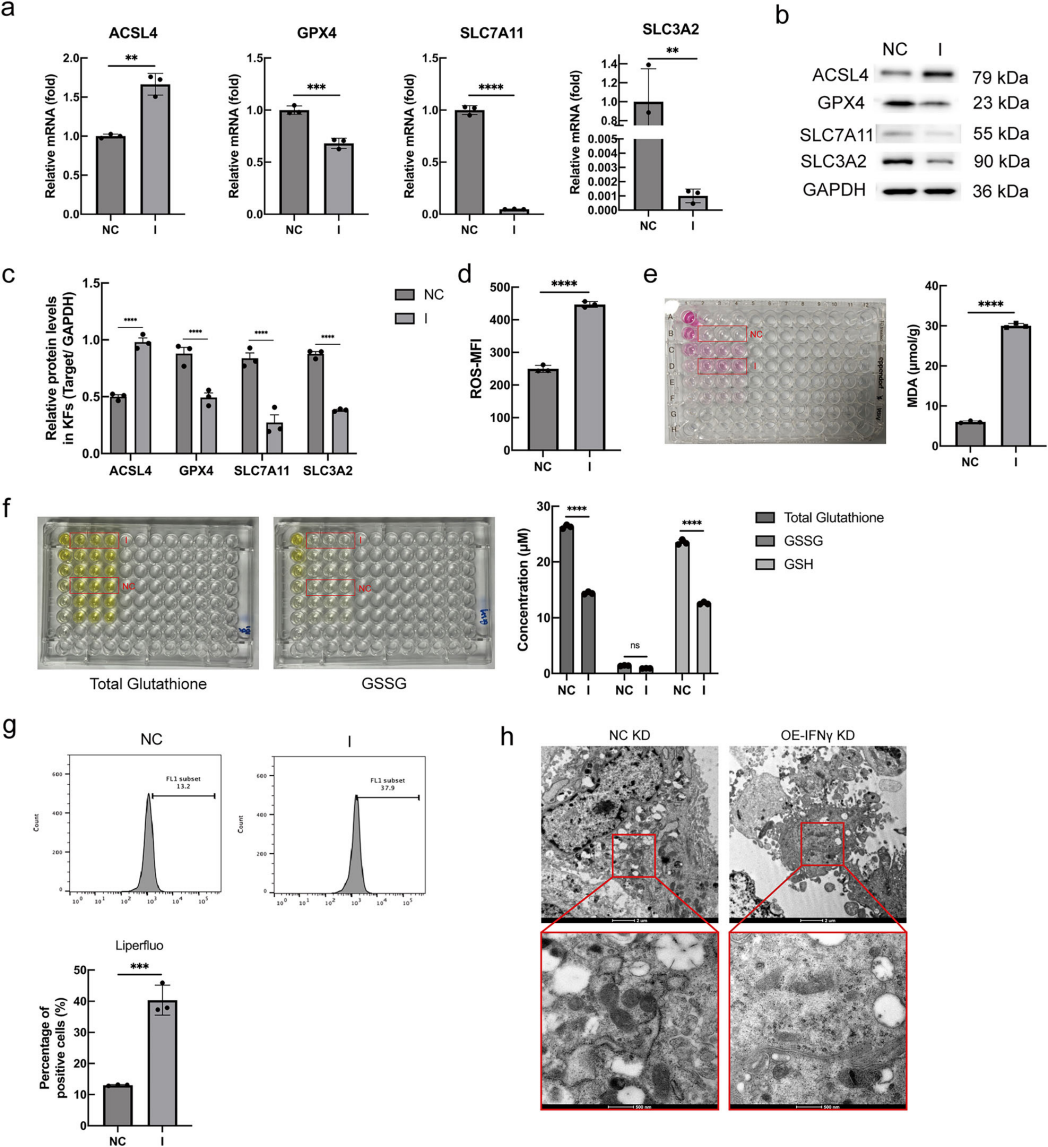
IFN-γ could induce ferroptosis in KFs. **a** Effect of IFN-γ on KFs’ gene expression which was detected by RT-qPCR. **b**, **c** Protein expression of ACSL4, GPX4, SLC7A11, and SLC3A2 was detected by western blot analysis. **d** The ROS detection was analyzed by flow cytometry. **e** The MDA concentration in the transfected KFs was detected using the MDA assay kit and measured with an enzyme marker. **f** The levels of total glutathione, GSH and GSSG in the transfected KFs. **g** Lipid peroxides were detected using a Liperfluo kit, and positively stained cells were presented. **h** Transmission electron microscopy images of transfected KFs. Scale bar = 2 μm and 500 nm (*n* = 3) (ns not significant; **P < 0.01, ***P < 0.001, ****P < 0.0001).

IFN-γ treatment was found to increase the mRNA and protein expression of ACSL4, a lipid metabolizing enzyme that promotes the formation of lipid peroxides, thereby triggering ferroptosis. In addition, IFN-γ inhibited the mRNA and protein expression of the two subunits of the system Xc^−^, namely, SLC7A11 and SLC3A2, and reduced the uptake of cystine by KFs; this, in turn, inhibited the downstream GPX4 signaling pathway (Fig. 2a–c). Overexpression of IFN-γ in KFs was followed by alterations in ferroptosis-related metabolites: The I group exhibited significantly greater levels of reactive oxygen species (Fig. 2d, *p* < 0.0001), as well as lipid peroxides and their metabolic breakdown product MDA (Fig. 2e, g, *p* < 0.0001, *p* < 0.001). Furthermore, the amount of GSH, a metabolic intermediate of ferroptosis (Fig. 2f, *p* < 0.0001), was decreased, but no significant change was found in the amount of GSSG (Fig. 2f, *p* > 0.05). This is suggestive of an enhanced response to ferroptosis as measured by the GSH/GSSG assay kit. Transmission electron microscopy was used to observe in more detail the role of IFN-γ in ferroptosis in KFs, and the results showed ed that the mitochondrial morphology of cells treated with the IFN-γ-overexpressing plasmid was altered, as evidenced by their smaller size and increase in their membrane density (Fig. 2h). All these results indicated that IFN-γ could induce KFs’ ferroptosis.

### IFN-γ-mediated inhibition of serpine2 transcription and translation in KFs

**Fig. 3:**
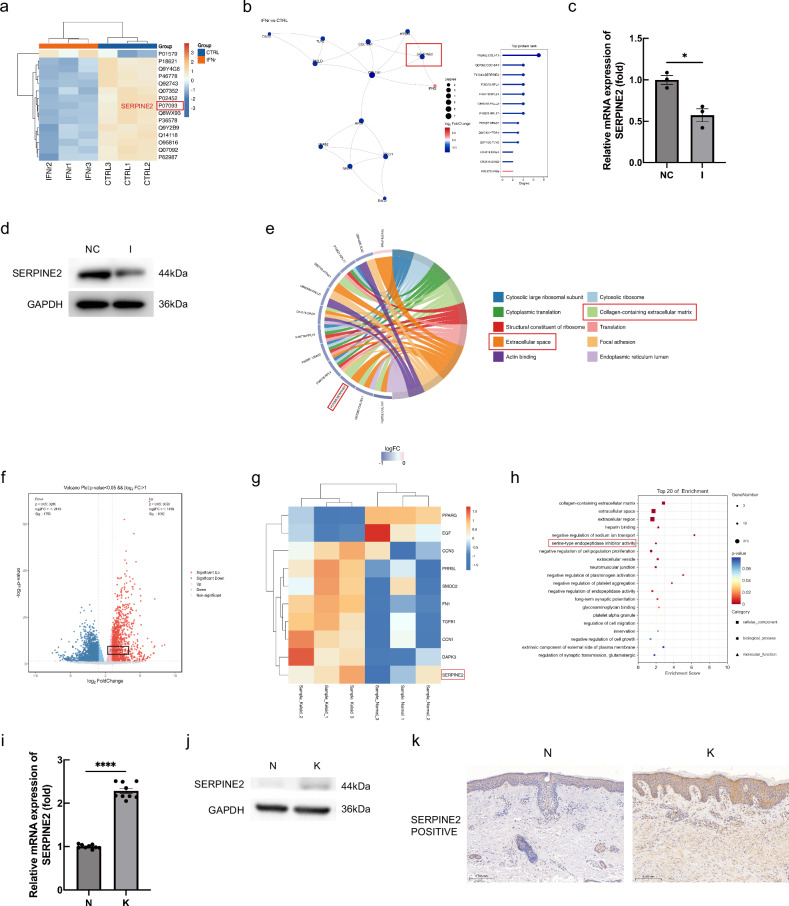
IFN-γ could inhibit the expression of serpine2 in KFs. **a** Clustering heatmap of TMT assay. **b** Proteomics results were analyzed, and a PPI network map was produced. **c** Gene expression was detected by RT-qPCR. (*n* = 3). **d** The protein expression of serpine2 was detected by western blot analysis. **e** GO enrichment analysis and chord diagrams results of TMT assay. **f** Volcano plots of RNA-seq. **g** Heat maps of RNA-seq results. **h** Top 20 functions or signaling pathways with significant differences in RNA-seq results. **i** RT-qPCR was performed to detect the gene expression level of serpine2 in NSFs and KFs. (*n* = 3). **j** Western Blot detection of protein expression levels of SERPINE2 in NSFs and KFs. **k** Immunohistochemically stained of serpine2. (*P < 0.05, ****P < 0.0001, scale bar = 200 μm).

In order to investigate the downstream signaling pathway by which IFN-γregulates ferroptosis in KFs, we performed TMT quantitative proteome sequencing of IFN-γrecombinant protein-treated (IFN-γ group) and untreated KFs (CTRL group). Subsequently, by having processed the raw data, we counted and identified differentially expressed proteins. A total of 14 proteins were down-regulated, and 1 protein was upregulated in terms of expression compared to the CTRL group, among which serpine2 (gene ID: P07093) belonged to one of the down-regulated proteins (Fig. 3a). We constructed a protein-protein interaction network (PPI) based on the results of sequencing analysis, which revealed that IFN-γcould directly inhibit the expression of serpine2 protein (Fig. 3b). We performed experimental validation with RT-qPCR and Western Blot experiments at the level of gene transcription and protein translation, respectively (Fig. 3c, d). To gain a deeper understanding of the functional roles of serpine2, we performed gene ontology (GO) enrichment analysis on the differentially expressed proteins, which showed that serpine2 was closely associated with collagen-rich extracellular matrix and extracellular space, and both of them were located in pathways significantly enriched in the IFN-γ group (Fig. 3e).

To further investigate whether serpine2 has a special significance in keloid formation, we selected three normal skin tissue samples and three keloid tissue samples for whole transcriptome sequencing. Compared with normal skin tissues, there were 1092 protein-coding mRNAs with up-regulated expression and 1750 protein-coding mRNAs with downregulated expression in keloid tissues (Fig. 3f), in which the expression of serpine2 gene was significantly increased (Fig. 3g). Further enrichment analysis revealed that serine-type endopeptidase inhibitor activity was among the top 20 significantly enriched biological functions or signaling pathways (Fig. 3h). To validate the above sequencing results, we used RT-qPCR and Western Blot experiments. The experimental data showed that both gene and protein expression levels of serpine2 were significantly elevated in KFs compared to normal skin fibroblasts (NSFs) (Fig. 3i, j). In addition, we performed immunohistochemical staining, and the results clearly showed that the density of serpine2-positive cells in keloid tissues was significantly higher than that in normal skin tissues (Fig. 3k). Taken together, serpine2 may be a key downstream gene in the process of IFN-γregulation of ferroptosis and related functional phenotypic changes in KFs.

### IFN-γ inhibits the proliferation, migration, and apoptosis of KFs induced by serpine2

**Fig. 4:**
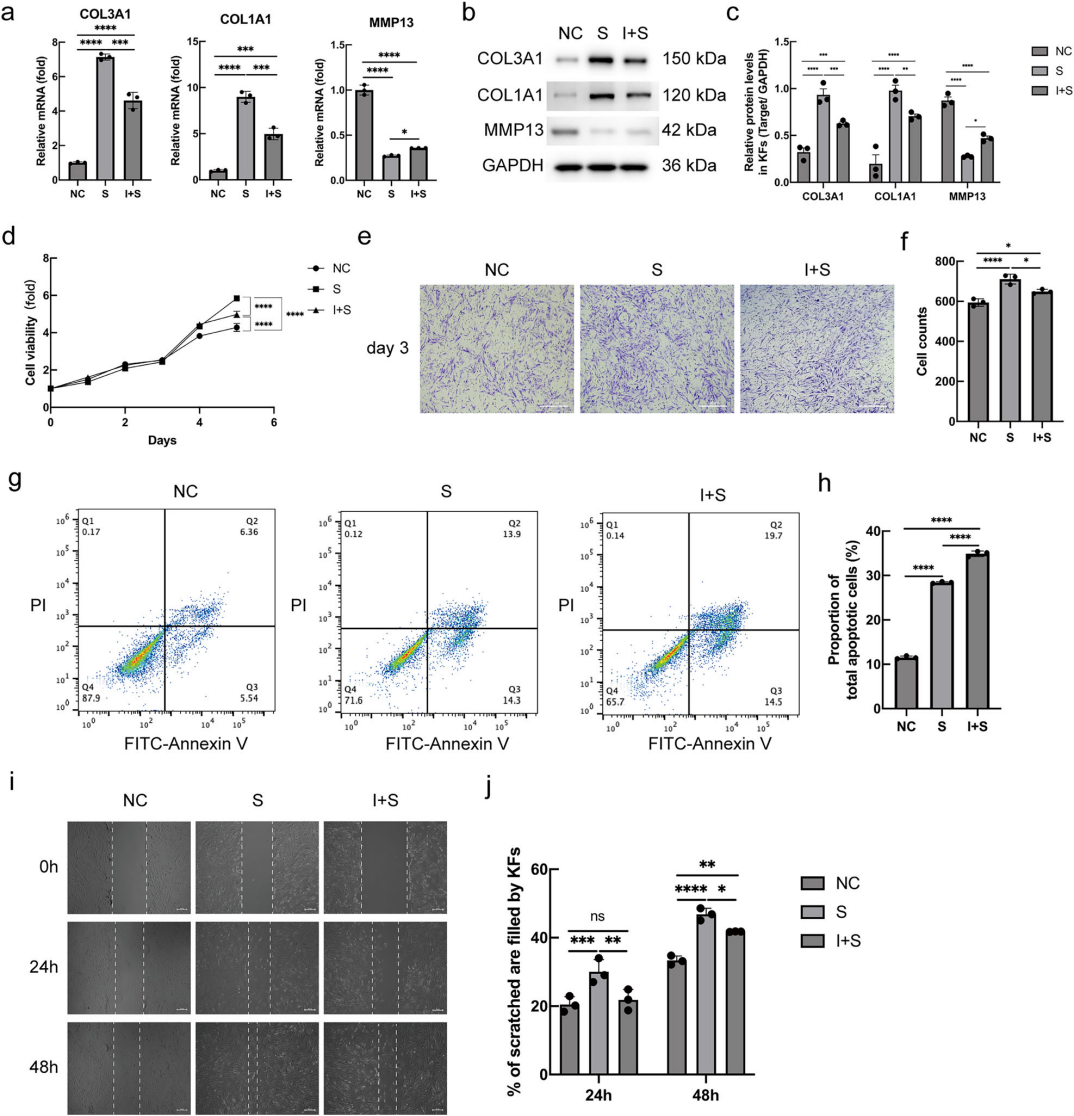
IFN-γ could inhibit KFs’ biological functions by inhibiting the expression of serpine2. **a**–**c** Effects of serpine2 and IFN-γ + serpine2 on the mRNA and protein expression of COL3A1, COL1A1, and MMP13 detected by RT-qPCR and western blot analysis, respectively. **d** Cell viability of KFs determined by the CCK8 assay on days 1, 2, 3, 4, and 5 after transfection. **e**, **f** The results of Transwell assay. **g**, **h** After incubation with Annexin V-FITC and PI of plasmid-transfected KFs, the flow cytometry was used to calculate the percentage of apoptotic cells in each group. **i**, **j** Wound healing images were taken 24 and 48 h after the medium was scratched. The experiments were conducted in triplicate and quantified using the FIJI software. (*n* = 3) (ns not significant; *P < 0.05, **P < 0.01, ***P < 0.001, ****P < 0.0001).

Based on the aforementioned findings, we proceeded to investigate whether serpine2 affects the expression of collagen and ECM proteins in KFs through an endogenous mechanism, thereby influencing cellular function. Additionally, we sought to ascertain whether IFN-γ exerts an inhibitory effect on the impact of serpine2 in KFs. After KFs were transfected with NC group, S group, and I + S group plasmids for 24 h, it was observed that the gene and protein levels of collagen types I and III (COL1A1 and COL3A1) were significantly greater in the S group-treated cells than in the NC group-treated cells (Fig. 4a–c). The I + S group-treated cells had significantly lower levels of collagen proteins than the S group-treated cells, though they were still higher than those in the NC group-treated cells (Fig. 4a–c). Thus, serpine2 promoted collagen synthesis and accumulation, whereas IFN-γ exerted a partial inhibitory effect on this process. It has been previously demonstrated that serpine2 can inhibit the expression of matrix metalloproteinase 13 (MMP13) [17], and this effect was verified in KFs in the present experiments (Fig. 4a–c). Subsequent experiments revealed that serpine2 was capable of promoting the proliferation (Fig. 4d), migration (Fig. 4i, j), and invasiveness (Fig. 4e, f) of KFs, and that IFN-γ was able to partially inhibit this effect of serpine2. However, overexpression of serpine2 promoted apoptosis of KFs, while overexpression of IFN-γ and serpine2 further enhanced apoptosis (Fig. 4g, h).

### IFN-γ regulated KFs’ ferroptosis through serpine2

**Fig. 5:**
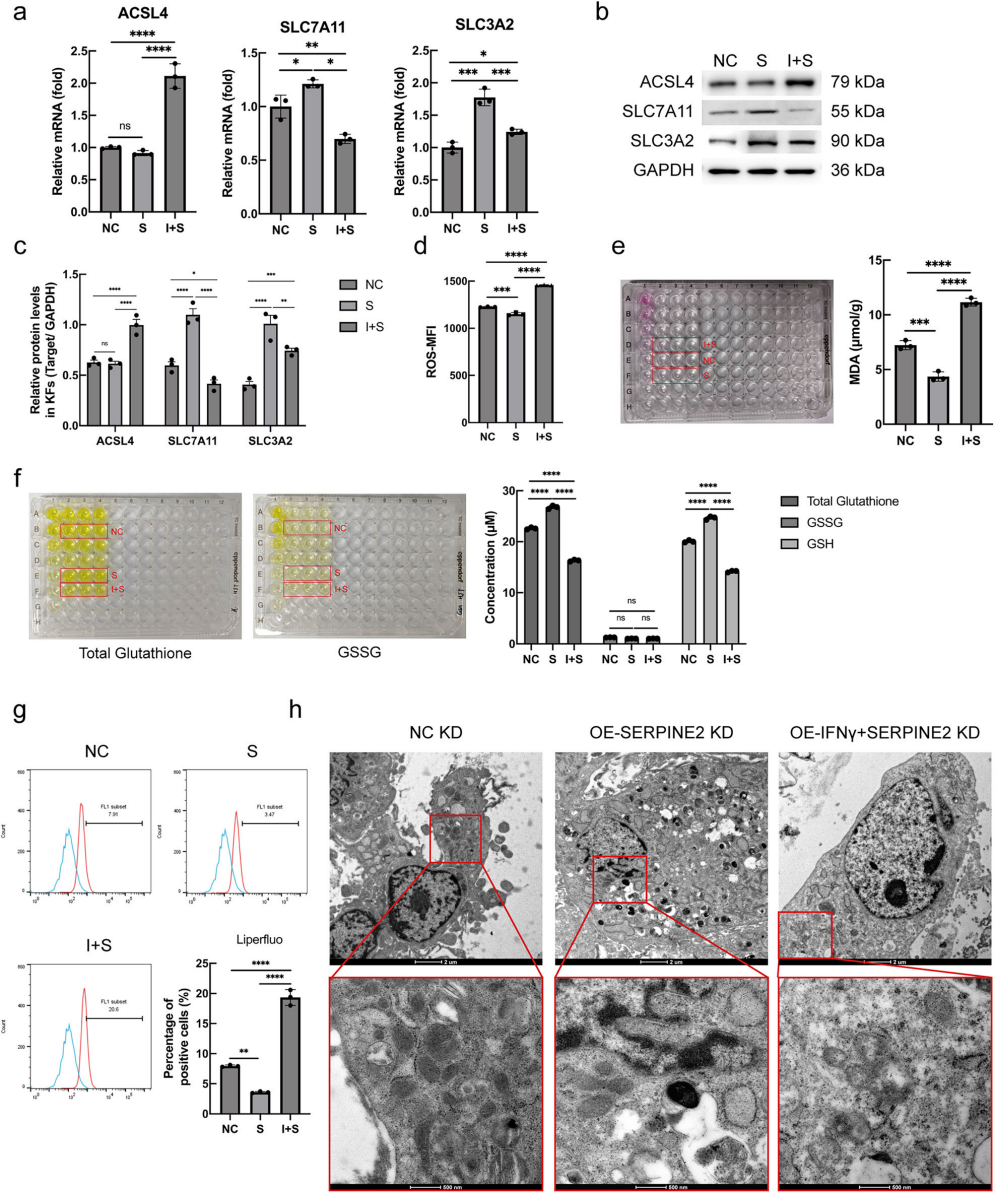
IFN-γ could regulate KFs’ ferroptosis through serpine2. **a**–**c** Effect of serpine2 and IFN-γ + serpine2 on the mRNA and protein expression detected by RT-qPCR and western blot analysis, respectively. **d** The results of ROS detection. **e** The MDA level in the transfected KFs. **f** The levels of total glutathione, GSH and GSSG in the transfected KFs were measured sequentially using the GSH/GSSG assay kit. **g** The results of Lipid peroxides. **h** Transmission electron microscopy images of transfected KFs. (*n* = 3) (ns not significant; *P < 0.05, **P < 0.01, ***P < 0.001, ****P < 0.0001, scale bar = 2 μm and 500 nm).

Whether IFN-γ could induce ferroptosis of KFs through serpine2 has been another problem for to solve. Overexpression of serpine2 did not affect ACSL4 transcription and translation but promoted the gene and protein expression of SLC7A11 and SLC3A2. Conversely, overexpression both of IFN-γ and serpine2 promoted ACSL4 gene and protein expression. However, simultaneous overexpression of IFN-γ and serpine2 (I + S group) inhibited the promotional effect of serpine2, although this group still exhibited significant differences in comparison to the NC group (Fig. 5a–c).

Overexpression of serpine2 in KFs also caused alterations in metabolites associated with the ferroptosis response. The levels of ROS (Fig. 5d, *p* < 0.001), lipid peroxides (Fig. 5g, *p* < 0.01), and the metabolic catabolite MDA (Fig. 5e, *p* < 0.001) were significantly lower in the S group than in the NC group but were significantly higher in the I + S group than in the NC or the S group (Fig. 5d, e, g). Moreover, the levels of GSH, a metabolic intermediate of ferroptosis, were higher in the S group and lower in the I + S group, in comparison to the NC group (Fig. 5f, *p* < 0.0001). However, there were no significant differences between the groups in terms of the GSSG levels (Fig. 5f, *p* > 0.05). Transmission electron microscopy showed that the mitochondrial morphology of the cells treated with serpine2-overexpression plasmids remained largely unchanged. However, in cells treated with IFN-γ- and serpine2-overexpression plasmids, a reduction in mitochondrial size, accompanied by an increase in membrane density and thickening of the cristae, was observed (Fig. 5h). Thus, serpine2 appears to inhibit ferroptosis in KFs; moreover, IFN-γ could inhibit this effect of serpine2.

### IFN-γ could induce KFs’ ferroptosis via inhibition of the expression of serpine2 in the in vivo model

**Fig. 6:**
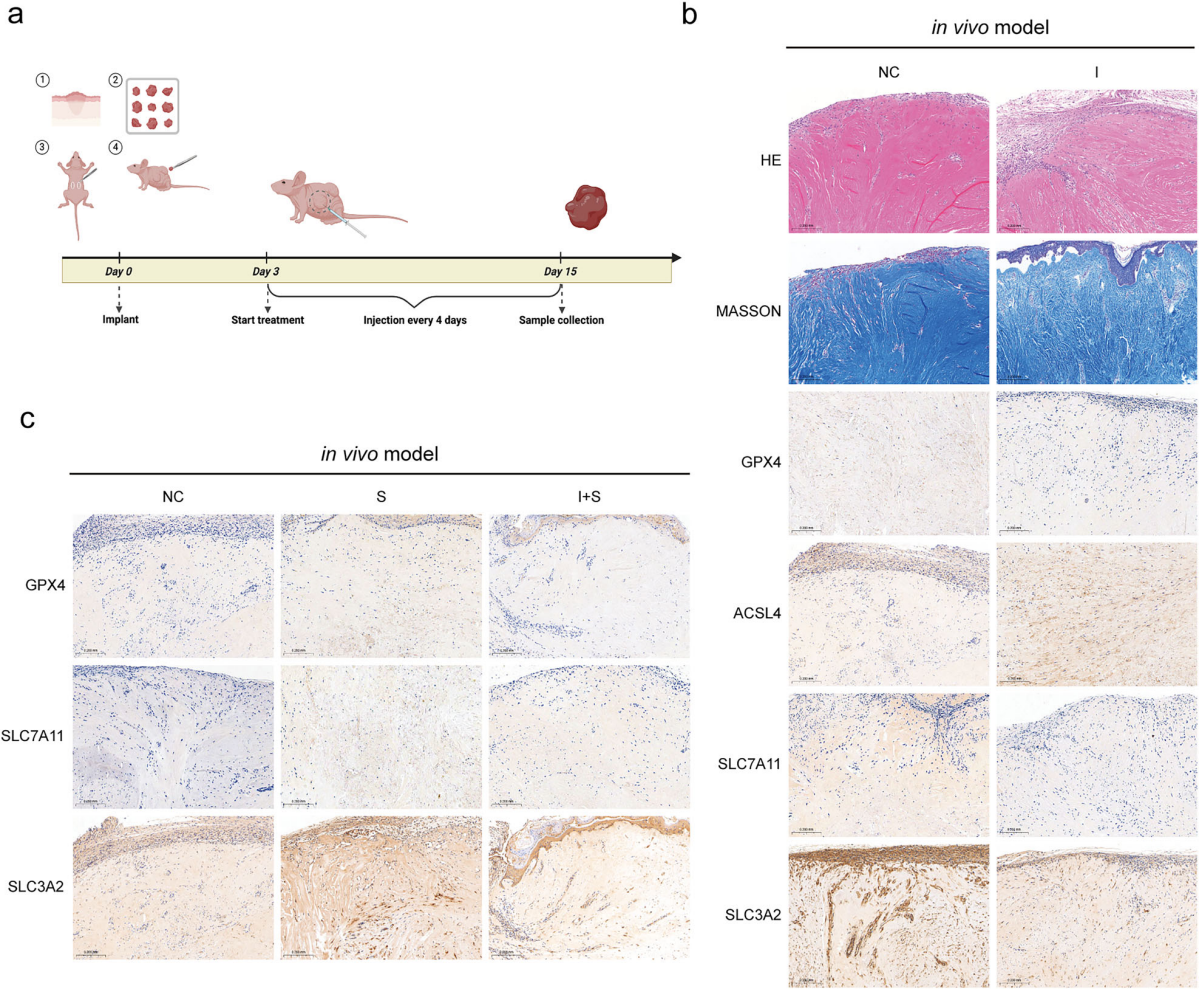
IFN-γ could regulate KFs’ ferroptosis through serpine2 in vivo. **a** Pattern diagram of in vivo experimental. **b** H&E staining, Masson’s trichrome, and IHC staining of in vivo keloid models with overexpressing of IFN-γ. **c** IHC staining of the GPX4, SLC7A11, and SLC3A2 proteins in in vivo keloid models with overexpressing both serpine2 and IFN-γ+serpine2 (**P < 0.01, ***P < 0.001, ****P < 0.0001, scale bar = 0.2 mm).

Administration of overexpression plasmids in an in vivo keloid model (established by embedding patient keloid samples subcutaneously in the backs of nude mice; Fig. 6a) resulted in overexpression of ACSL4 that was promoted by IFN-γ, while the expression of GPX4, SLC7A11, and SLC3A2 was inhibited (Fig. 6b). Conversely, overexpression of serpine2 had the opposite effect: that is, it promoted the expression of GPX4, SLC7A11, and SLC3A2, and this was partially inhibited by IFN-γ (Fig. 6c). These findings were validated through immunohistochemical analysis (Fig. 6b, c). Furthermore, we conducted H&E and Masson staining of keloid xenografts from group NC and group I (Fig. 6b), and the results implied that overexpression of IFN-γ caused a reduction in collagen deposition and accumulation, as evidenced by the transition from dense and coarse collagen bundles to fine and shallow collagen bundles. To make the argument more rigorous, we also conducted a DFO protective control experiment using an in vivo animal model to demonstrate this point more strongly (Fig. S5). Thus, the in vivo results are consistent with the in vitro cellular observations and demonstrate that IFN-γ can induce ferroptosis in KFs through the inhibition of serpine2.

## Discussion

The findings of the present study demonstrate the antiproliferative and antifibrotic effects of IFN-γ in keloid tissue and the related mechanism involving the inhibition of collagen synthesis and α-SMA expression in KFs. This mechanism of reduced collagen synthesis and α-SMA expression corroborates reports from as early as 2002. In fact, the *International Clinical Recommendations on Scar Management* suggested that IFN-γ could promote collagen degradation and suppress serum transforming growth factor-β levels, making it a valuable tool for enhancing keloid treatment and preventing recurrence after surgical intervention [6]. Thus, we believe that IFN-γ holds great promise as an immunomodulator, and we plan to explore additional mechanisms and downstream pathways involved in its therapeutic potential in keloid treatment.

Several studies have explored the role of IFN-γ in regulating ferroptosis and have shown that IFN-γ derived from CD8+ T cells bind to the ACSL4 promoter, inducing ferroptosis in tumor cells [15]. With regard to the mechanism, the JAK1-2/STAT1/SLC7A11 signaling pathway has been implicated in mediating IFN-γ-induced ferroptosis in retinal pigment epithelial cells [18]. This process is characterized by increased levels of ROS and lipid peroxides accompanied by depletion of GSH, which are key markers of ferroptosis. Our study demonstrated that overexpression of IFN-γ in KFs led to similar cellular changes. They indicate that IFN-γ may inhibit cystine uptake by upregulating ACSL4 and downregulating SLC3A2 and SLC7A11, thus resulting in greater GSH depletion, enhanced ROS production, and reduced GPX4 levels, and rendering KFs more susceptible to ferroptosis. We also demonstrated that KFs were more sensitive to erastin, a known inducer of ferroptosis, after exposure to IFN-γ, with significantly higher levels of ROS than the single-drug group (Figure S4). We suggest that the mechanism of IFN-γ-induced ferroptosis involves two main aspects. On the one hand, IFN-γ is able to down-regulate the expression of GPX4, which reduces the cellular scavenging capacity of lipid peroxides, leading to a weakened antioxidant capacity, which in turn triggers a spontaneous ferroptosis response. On the other hand, IFN-γ also induced ferroptosis by inhibiting the function of System Xc^−^, a key pathway for cellular uptake of cystine for the synthesis of GSH, which, as an important antioxidant, is essential for protecting cells from oxidative stress damage. When System Xc^−^ function is inhibited, cystine uptake is reduced, leading to insufficient GSH synthesis and a subsequent decrease in antioxidant capacity, which may trigger ferroptosis. Notably, these two mechanisms are not mutually exclusive, and they may function simultaneously or separately in different cell types or conditions. In addition, the exact mechanism of IFN-γ-induced ferroptosis may involve more complex signaling pathways and molecular interactions, which need to be further investigated to clarify. RSL3, a known inhibitor of GPX4, causes intracellular lipid peroxidation by inhibiting GPX4 activity, thereby inducing ferroptosis, and this mechanism of action has been validated by us (Fig. S2). We also performed protective control experiments using Fer-1, an inhibitor of ferroptosis and confirmed that ferroptosis is a specific death pathway for IFN-γ action in KFs (Fig. S3). Put together, these observations imply that the therapeutic effect of IFN-γ in keloids is related to its ability to promote ferroptosis in KFs.

As part of the serine protease inhibitor family [19], serpine2 can inhibit various serine proteases, including thrombin, urokinase, fibrinolytic enzymes, and trypsin [20, 21], and plays a crucial role in physiological processes such as coagulation, fibrinolysis, and inflammation [22]. Serpine2 overexpression can promote fibronectin synthesis and regulate ECM protein expression in normal lung fibroblasts to contribute to the development of idiopathic pulmonary fibrosis [23, 24]. Furthermore, exogenous serpine2 enters cardiac fibroblasts via endocytosis, activating the ERK1/2 and β-catenin signaling pathways and, thus, enhancing collagen production in fibroblasts and inducing cardiac fibrosis [25, 26]. These findings highlight the potential role of serpine2 in fibrotic diseases. Similarly, our study demonstrated that exogenous serpine2 overexpression enhances collagen production in KFs, thus supporting key cellular functions such as proliferation, migration, and invasion. Additionally, we found that serpine2 significantly inhibited the expression of MMP13 in KFs. However, we also observed that serpine2 overexpression induced apoptosis in KFs. This could be explained by the effect of the overexpression plasmid transfection process on cell viability. Nonetheless, this observation provides compelling evidence for the role of serpine2 in promoting the functions of KFs. Further, based on our observation that serpine2 is the most significantly downregulated gene in response to exogenous IFN-γ overexpression, it is reasonable to hypothesize that serpine2 plays a key role in the IFN-γ-induced ferroptosis pathway in KFs. However, further investigation into this hypothesis is warranted.

Our results demonstrated that exogenous overexpression of serpine2 upregulated SLC7A11 and SLC3A2 expression, enhancing cystine transport via the system Xc^−^ antiporter. This led to reduced GSH depletion, decreased intracellular ROS levels, increased GPX4 activity, and a subsequent reduction in lipid peroxides and their byproduct MDA. All of these processes are known to be associated with ferroptosis.

**Fig. 7:**
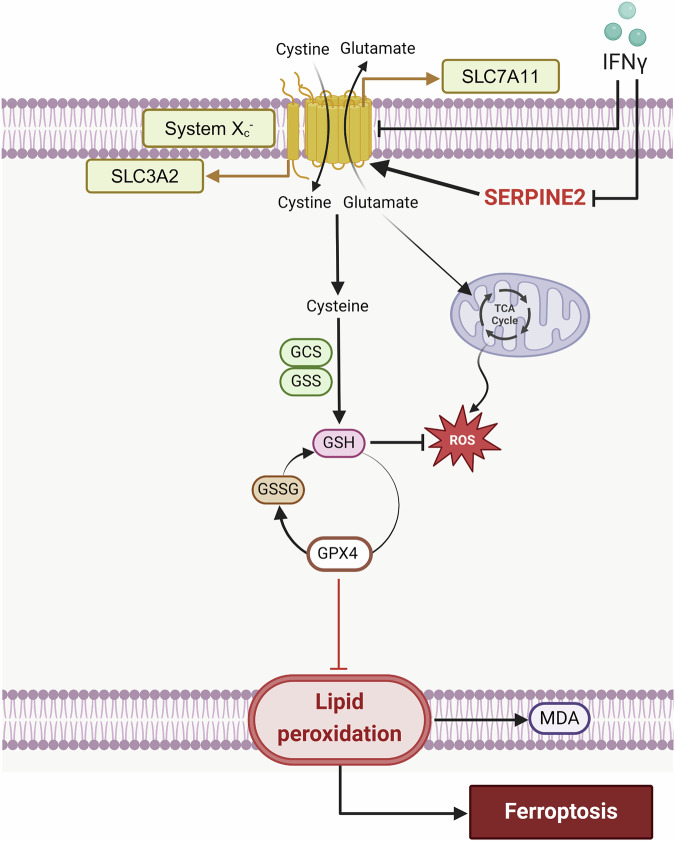
Schematic representation of IFN-γ induces keloids’ ferroptosis through inhibiting serpine2.

In the experimental group where both IFN-γ and serpine2 were overexpressed (I + S group), the ferroptosis-promoting effect was significantly stronger than that in the serpine2-only group (S group), while the ferroptosis-inhibiting effect was notably lower than that in the S group. Further, gain-of-function experiments with serpine2 and IFN-γ revealed that exogenous serpine2 could suppress ferroptosis in KFs, while IFN-γ inhibited serpine2, thereby promoting ferroptosis (Fig. 7). Based on these findings, we hypothesize that IFN-γ may enhance the sensitivity of KFs to ferroptosis by inhibiting serpine2 expression. In line with this notion, recent studies have shown that inhibiting the serpine2-EGFR axis can effectively suppress the metastasis and invasion of hepatocellular carcinoma cells, while also enhancing their sensitivity to the target drug sorafenib [27]. Further, sorafenib, a SLC7A11 antagonist, did not directly induce ferroptosis but, instead, blocked cell proliferation and induced cell death through other pathways [28]. This implies that IFN-γ may induce cell death in KFs through pathways other than ferroptosis. These complex interactions between the cell death mechanisms of IFN-γ need to be further explored to understand better its therapeutic effects in fibrotic diseases.

It is important to acknowledge the inherent limitations of our study. The results have only been validated in primary KFs and in previously established, relatively reliable, in vivo models. However, these models do not fully capture the complexity and characteristics of keloids as they occur in vivo. Additionally, there is currently a lack of clinical or preclinical targeted therapies specifically aimed at serpine2. Nevertheless, recent advancements in targeted therapeutic strategies, such as nanocarrier-encapsulated siRNAs, present promising avenues for further investigation. Our future research will focus on developing more accurate in vivo models of keloids and exploring the specific molecular targets involved in the interaction between IFN-γ and serpine2.

## Conclusion

We have identified serpine2 as a gene that inhibits ferroptosis in KFs by promoting the expression of the system Xc^-^ antiporter, which in turn enhances collagen and ECM synthesis and supports KF proliferation, migration, and invasion. Additionally, our findings show that IFN-γ not only directly induces ferroptosis in KFs but also enhances their sensitivity to ferroptosis by inhibiting the synthesis of SLC7A11 and SLC3A2 through the downregulation of serpine2. Thus, the serpine2-system Xc^−^-axis may present a promising therapeutic target for the treatment of keloids.

## Materials and methods

### Keloid tissue and normal skin tissue sampling

We obtained 37 normal skin tissue and active keloid samples from 23 patients (patient information provided in Supplementary Table S1) who had not yet received keloid treatment from Shanghai Ninth People’s Hospital. Primary human KFs and normal skin fibroblasts were isolated from keloid tissue and normal skin tissue, respectively. We received approval for the use of the patient samples from the Ethics Committee of the Ninth People’s Hospital of Shanghai Jiaotong University School of Medicine. The experimental protocols were in accordance with the Declaration of Helsinki, and the patients provided their informed consent for the use of their samples.

### Isolation of KFs

Surgically excised keloid tissue was obtained and placed in serum-free DMEM containing 30% antibiotic-antimycotic for 30 min, followed by three oscillatory rinses in a 0.25% chloramphenicol solution to clean and disinfect. After the removal of excess fat, the surface was finely drawn and gridded for cutting and then immersed in a 0.3% dispase neutral protease solution for overnight treatment at 4 °C. On the following day, the epidermis was peeled off by gentle friction with sterile gauze to obtain the dermal tissue, which was finely cut into small 1 mm^3^ pieces and rinsed with PBS containing 1% penicillin–streptomycin with oscillation. The tissue block was placed in 3–4 times the volume of 0.2% NB4 collagenase solution prepared with low sugar DMEM and digested at 37 °C for 6–8 h to obtain primary fibroblasts. The digested solution was filtered through sterile gauze to remove the undigested tissue mass, centrifuged (1500 rpm, 5 min) twice to remove the supernatant, resuspended with culture medium, transferred to a 10-cm petri dish, shaken well, and then incubated in a constant temperature CO_2_ incubator.

### Animal models

Male BALB/C nude mice aged 4–6 weeks were purchased from Shanghai Bicaikewing Biotechnology Co. Ltd. (Shanghai, China). The mice were housed in an SPF-grade environment at 24 °C and 50% humidity. The procedures adhered to the Guidelines for the Care and Use of Laboratory Animals approved by the Ethics Committee for Animal Experimentation of the School of Medicine of Shanghai Jiao Tong University. At the end of the experiment, the mice were anesthetized by carbon dioxide inhalation and sacrificed according to the guidelines of the Canadian Council on Animal Care (CCAC). The animal experiments received the approval of the Ethics Committee of the Ninth People’s Hospital of Shanghai Jiaotong University School of Medicine (approval No. SH9H-2021-A371-SB).

### Packaging and transfection of overexpression plasmids

IFN-γ (EX-A0177-M02) and SERPINE2 (EX-T2631-M02) overexpression plasmids and an empty negative control plasmid (EX-NEG-M02) were synthesized by Guangzhou GeneCopoeia^TM^ Ltd. (China). The primers for the construction of the overexpression vectors are shown in Table S2. The transfection groups included 2 μg of IFN-γ-overexpression plasmid (I group), 2 μg of SERPINE2 overexpression plasmid (S group), 2 μg of IFN-γ overexpression plasmid and 2 μg of SERPINE2 overexpression plasmid (I + S group), and an empty-load negative control plasmid (NC group). These plasmids/plasmid combinations were dissolved in 150 µl of serum-free DMEM containing 5 μl of Lipo3000. The medium was mixed well and allowed to stand for 20 min at 28°C. Then, 300 µl of the DNA liposome complex obtained from the different groups was added to six-well plates containing KFs at a density of 60–70% and incubated for 24–48 h to allow for transfection. The transfected cells were collected for further experiments.

### In vivo keloid model

The nude mouse subcutaneous graft scar tissue block model was based on Professor Tao Zan’s published model [29, 30]. We randomly divided 12 mice into four groups of three mice each. The keloid tissue blocks were cut into 5 mm × 5 mm × 5 mm pieces, with one piece of tissue each implanted subcutaneously on the left and right side of the back. Then, 5 μg each of the NC group, I group, S group, and I + S group plasmids was dissolved in 10 μl of EntransterTM-in vivo (18668-11-2; Engreen Biosystem Co. Ltd.), diluted to 50 μl in sterile saline, and injected into each tissue implant every 4 days thereafter. On day 15, one part of the treated implanted tissue block was placed in a 4% paraformaldehyde solution for overnight fixation for further histological analysis, and the other part was immediately frozen in liquid nitrogen for protein extraction.

### Reverse-transcription quantitative PCR

KFs of each group after transfection of the experimental design were collected, and RNA was extracted using the EZ-press RNA Purification Kit (B004DP; EZBioscience, MN, USA), and after lysis, mixing with anhydrous ethanol, centrifugation, washing, and air-drying, RNA samples were obtained by centrifugation with the addition of eluent. After detecting the sample concentration and OD260/OD280 ratio to ensure that it was within the range of 1.8–2.0, the RNA was reverse transcribed using the Color Reverse Transcription Kit (A0010CGQ; EZBioscience, MN, USA), and cDNA was obtained. Specific primer sequences designed based on the information in the NCBI Gene database (the primer sequences are listed in Table S3) were synthesized by Shanghai Shenggong Biotech. Subsequently, the qPCR system was prepared for real-time fluorescence quantitative PCR reaction (95 °C pre-denaturation for 5 min, followed by 40 cycles of 95 °C for 10 s and 60 °C for 30 s) using the 2× Color SYBR Green qPCR Master Mix (ROX2 plus) (A0012-R2; EZBioscience, MN, USA) with 20-fold diluted cDNA as template. Finally, after three repetitions of the experiment, the relative expression levels of the target genes were statistically analyzed using the 2^−ΔC(t)^ method with GAPDH as an internal reference.

### Western blotting analysis

Firstly, the protease inhibitor was mixed into the RIPA lysate in the ratio of 1:100, and each well of the six-well plate was lysed by adding 125 μl of the mixture on ice and centrifuged to take 105 μl of the supernatant as the protein samples, and the absorbance was determined, and the protein concentration was calculated by adding the incubation of liquid A and liquid B. Subsequently, the loading buffer was added to denaturate the samples and stored. Then, electrophoresis gel was prepared, electrophoresis solution was prepared, and samples and molecular weight standards were added for electrophoretic separation. After electrophoresis, the PVDF membrane was cut and activated, and a membrane transfer solution was prepared for wet transfer to the membrane, which was closed, rinsed with TBST, incubated with the primary antibody overnight, then recovered the primary antibody and incubated with secondary antibody the next day, and then rinsed again, and then mixed with solution A and B (BeyoECL Moon detection kit, Beyotime, China) to form a developer solution for developing and photographing (see Table S4 for antibodies and Fig. S6 for the raw western blots).

### Cell proliferation assay

For assessing cell proliferation with the cell counting kit-8 (CCK-8), 96-well plates were seeded at 5 × 10^3^ post-transfected KFs per well according to the CCK8 User Manual (Dojindo, Japan). We performed tests daily, from day 1 to day 5 after treatment. Measurements were performed using a microplate reader (Biotek, Vermont, USA) with a peak absorption wavelength of 450 nm. The obtained OD values of each well were subtracted from the OD values of the blank wells, and then the average of all the well values on day 0 of each group was calculated and homogenized for subsequent graphing and analysis.

### Transwell cell invasion assay

First, 300 µl of DMEM medium containing 20% fetal bovine serum was added to the lower chamber of 24-well Transwell membrane filter inserts (Corning Costar, USA). Next, transfected cells were diluted to 5 × 10^5^ cells ml with serum-free DMEM medium, and 100 µl of the solution was seeded in each inner chamber. The invasion cycle was 72 h. The chambers were washed with phosphate-buffered saline and fixed with 4% paraformaldehyde solution for 15 min. Cells that did not penetrate the filters were wiped out, and cells that had adhered to the lower surface were treated with 0.4% crystal violet stain. The lower layer of the polycarbonate membrane was observed under a microscope (Leica EZ4) and photographed. The cells were counted with the Image J software (Media Cybernetics, MD, USA).

### FITC Annexin V apoptosis detection assay

Collect 2 × 10^6^ untreated keloid fibroblasts, wash and centrifuge twice with pre-cooled PBS, discard the supernatant, dilute 5× Binding Buffer with ddH_2_O to 1× Binding Buffer, add it to the cell precipitation to 1.5 ml, and then aliquot it into 3 tubes, of which 1 tube was the blank control group, and 2 tubes were the single-stained group. Add 5 μl Annexin V-FITC or 10 μl PI to the single-stained samples (BD Pharmingen, NY, USA), and incubate for 30 min at room temperature with no light. The voltage of the FSC, SSC, and fluorescence channel was adjusted with the blank tube first, and then the compensation of the fluorescence channel was adjusted with the single-stained tube. Collect 1 × 10^6^ KFs after transfection, wash with pre-cooled PBS, and then centrifuged to collect the cell precipitate. Take 500 μl of 1× Binding Buffer to resuspend the cells, and then add 5 μl of Annexin V-FITC and 10 μl of PI and incubate for 30 min at room temperature, avoiding light, before counting the percentage of apoptotic cells on the machine.

### Cell migration assay

Post-transfected cells (2 × 10^5^) were cultured in each well of a 6-well culture plate. Subsequently, a scratch was made with a 200 μL pipette tip perpendicular to the bottom of the plate on each confluent monolayer of cells. Photographs of each scratch were taken immediately under a microscope (Zeiss, Germany) and again after 24 and 48 h, with a change of medium. The distance between the scratches relative to the edges (defined as the Gap) was estimated using Image J (Media Cybernetics, MD, USA) with the following formula: cell migration rate (%) = (Gap0 h − Gap24 h)/Gap0 h × 100%.

### ROS assay

Following a 24-h incubation period with overexpression plasmids, the cells were trypsinized, collected, and evaluated with a Reactive Oxygen Species Assay Kit (Beyotime, China). Cells were incubated with the Reactive Oxygen Detection Kit at 37°C for 30 minutes before being analyzed by flow cytometry.

### Lipid peroxidation assay

Cells were treated according to the instruction manuals for the Malondialdehyde (MDA) Detection Kit (Beyotime, China) and the Liperfluo Cellular Lipid Peroxidation Kit (Dojindo, Japan). The level of MDA was presented as nmol/mg protein, and the cells were analyzed using flow cytometry to detect the lipid peroxide levels in live cells.

### Reduced glutathione assay

Briefly, post-transfected cell samples were processed according to the manual of the glutathione (GSH) Assay Kit (Beyotime, China), and absorbance was measured with the enzyme marker A412. The GSH content was calculated from the measured total glutathione content and the oxidized glutathione (GSSG) content as follows: GSH = Total Glutathione − GSSG × 2. The GSH level was expressed as nmol/mg protein.

### Transmission electron microscopy

At 48 h after plasmid transfection, cells were imaged with a transmission electron microscope (HITACHI HT7800).

### RNA sequencing and data processing

RNA was extracted from fresh normal skin tissue and keloid tissue samples using the Trizol reagent. Eukaryotic mRNA was enriched from the total RNA content by magnetic beads with Oligo (dT) and randomly cut into fragments of appropriate lengths with a fragmentation buffer, which was used for their transformation into cDNA libraries. Their abundance and size distribution were verified to ensure that the library quality met the sequencing requirements. The qualified RNA-seq library was then loaded onto a high-throughput sequencer (Illumina NovaSeq 6000) for sequencing, and the raw sequencing data file in the FASTQ format was generated with the sequencing-by-synthesis technology. The sequencing data were subjected to quality control, sequence alignment, and transcript assembly and quantification, and differentially expressed genes were identified using the DESeq2 algorithm, with a false discovery rate < 0.05 and | log2(fold change) | ≥ 1 used as criteria. Sequencing was performed by Lu Ming Biotech (Shanghai, China).

Gene Ontology (GO) and Kyoto Encyclopedia of Genes and Genomes (KEGG) analyses were conducted with the Ouyi Cloud (https://cloud.oebiotech.com/), and *p* < 0.05 was considered to indicate significant differences. A cluster heat map, a GO-KEGG bubble map, and a volcano plot were created using the Ouyi Cloud platform.

### Tandem mass tag-tagged quantitative proteome sequencing

Proteins were extracted from IFN-γ-treated and non-IFN-γ-treated keloid fibroblasts using RIPA lysate. Protein concentration was determined using the Bradford assay for further quality control. The purified proteins were digested with trypsin to generate peptides suitable for mass spectrometry analysis. Each sample was labeled according to the ratio of tandem mass tag (TMT) marker: peptide, which was set as 5:1. Liquid-phase separation was performed using an UltiMate™ 3000 Binary Rapid Separation System (Thermo Fisher Scientific, San Jose, CA), and the resulting peptides were ionized by a nanoESI source with a mass spectrometer, Orbitrap Exploris™ 480 (Thermo Fisher Scientific, San Jose, CA) and detected in the data-dependent acquisition mode. Sequencing was performed by Lu Ming Biotech (Shanghai, China). Protein–protein interaction (PPI) networks were generated with the Ouyi Cloud platform (https://cloud.oebiotech.com/), and *p* < 0.05 was considered to indicate significant differences.

### Hematoxylin-eosin staining, Masson staining, and immunohistochemical analyses

For standard analyses, implanted keloid tissue blocks extracted after 15 days from the animal models were stained with hematoxylin and eosin (H&E) and Masson. SLC7A11, SLC3A2, GPX4, and ACSL4 antibodies were diluted as recommended by the product instructions and incubated with the keloid sections. Six randomly selected fields of view were selected for qualitative and quantitative evaluation of the target antigens in the tissue. The antibodies used are listed in Table S4.

### ELISA to detect the promoting effect of overexpression plasmid transfection on IFN-γ

80%–90% fused 10 cm Petri dishes of KFs were trypsin digested and seeded in six-well plates. Cells were grouped after stable wall affixation: blank group (adding medium mixture containing only lipo3000), negative control group (adding 2 μg of empty plasmid and lipo3000), and experimental group (adding 2 μg of IFN-γ overexpression plasmid and lipo3000). The medium was changed after 24 hours and washed with PBS, and the medium was changed for day 0 of the experiment. The supernatant was collected on days 1–3 for ELISA to detect IFN-γ expression.

Using the 48 T ELISA kit (CUSABIO, China), double-antibody sandwich method: dilute the standard to 8 concentration gradients, set up the standard wells, the sample wells to be tested, and the blank control wells, and add the corresponding solutions. Add biotin-labeled antibody, incubate at 37 °C for 30 min, and wash 5 times. Add the colorant and incubate for 10 min at 37 °C, then add the termination solution. Measure the OD value at 450 nm and calculate the concentration of IFN-γ in the samples to be tested by plotting the standard curve.

### Double staining of living and dead cells

In order to deeply investigate whether ferroptosis is a specific cell death pathway in IFN-γ-induced death of KFs, we designed the following experimental subgroups to treat KFs: the NC group (transfected with a blank vector plasmid), the I group (transfected with an IFN-γ plasmid alone), and the I+Fer-1 group (transfected with an IFN-γ plasmid at the same time and added with 2 μM of Fer-1). Subsequently, we stained the treated cells according to the operating instructions of the Calcein-AM/PI Double Staining Kit (Yeasen, China). Finally, to further analyze the effect of ferroptosis inhibitor Fer-1 on IFN-γ-induced cell death, observations were made under a fluorescence microscope to differentiate and accurately count live (green fluorescence) and dead cells (red fluorescence).

### Statistical analysis

In this study, we used SPSS software (version 19.0) as the main tool for statistical analysis. All results are reported as mean ± standard deviation (SD). To assess the differences between the two groups of data, we applied the *t*-test method. For comparisons involving multiple groups of data, we instead used a one-way analysis of variance (ANOVA), supplemented by appropriate post-hoc multiple comparisons means to further parse out differences between the data. For the CCK-8 cell proliferation assay in this study, we used a curve-fitting model and performed an *F*-test to compare kinetics. Differences were considered statistically significant at *p* < 0.05.

## Supplementary information

SUPPLEMENTARY TABLES

SUPPLEMENTARY FIGURES

SUPPLEMENTARY FIGURE LEGENDS

Western Blot Original Data

Raw and differential data from proteomics sequencing

RNA-seq raw data

## Data Availability

The data that support the findings of this study are available from the corresponding author upon reasonable request.
