# Supplementary material for: IFN-γ could induce ferroptosis in keloid fibroblasts by inhibiting the expression of serpine2
Source: Cell Death Discov. 2025 May 5;11:217. doi: 10.1038/s41420-025-02401-3 (PMC12053758; doi:10.1038/s41420-025-02401-3)
Supplement: Supplementary file 1 — SUPPLEMENTARY TABLES [file 41420_2025_2401_MOESM1_ESM.docx]

**SUPPLEMENTARY TABLES**

**IFN-γ could induce ferroptosis in keloid fibroblasts through inhibiting the expression of serpine2**

Jingyan Huang^1*^, MD, Yu Shun^2*^, MD, Jing Luo^1^, MD, Xusong Luo^1^, PhD, Jun Yang^1#^, PhD, MD, Xiuxia Wang^1#^, PhD

**Table S1.** Demographic data of keloid and normal skin samples used in this study

| **Sample**  **No.** | **Gender** | **Ethnic**  **Background** | **Age** | **Site of Specimen Collected** | **The Size of the Specimens**  **(cm^2^)** |
| --- | --- | --- | --- | --- | --- |
| KS1 | F | Asian | 25 | Chest | 5×2 |
| KS2 | M | Asian | 28 | Chest | 5×4; 3×3 |
| KS3 | M | Asian | 31 | Chest | 2×4; 3×3.5 |
| KS4 | M | Asian | 19 | Chest | 4×2 |
| KS5 | F | Asian | 43 | Chest | 4.5×3.5; 2×4 |
| KS6 | M | Asian | 21 | Chest | 3×5 |
| KS7 | F | Asian | 48 | Chest | 6.5×3.5; 2×3 |
| KS8 | F | Asian | 32 | Chest | 2×1.5; 3×3; 2×1 |
| KS9 | M | Asian | 25 | Chest | 6×4.5 |
| KS10 | M | Asian | 24 | Chest | 2.5×5.5; 4×2 |
| KS11 | F | Asian | 52 | Chest | 3×2; 2.5×4 |
| KS12 | F | Asian | 34 | Chest | 4×5 |
| KS13 | F | Asian | 53 | Chest | 9.5×2; 2×2 |
| KS14 | M | Asian | 25 | Chest | 6×1.5; 3×2 |
| KS15 | M | Asian | 37 | Chest | 5×3.5; 2×4 |
| KS16 | F | Asian | 29 | Chest | 5×3 |
| KS17 | F | Asian | 49 | Chest | 4.5×2.5 |
| KS18 | M | Asian | 16 | Chest | 2.5×3 |
| KS19 | M | Asian | 37 | Chest | 3.5×2 |
| NS20 | F | Asian | 34 | Abdomen | 11×7; 6.5×5 |
| NS21 | M | Asian | 12 | Scalp | 6×2.5; 5×1.5 |
| NS22 | M | Asian | 6 | Scalp | 3.5×3; 4.5×1.5 |
| NS23 | M | Asian | 8 | Lower limbs | 5.5×3 |

**Note:** Samples KS17-KS19 were used for keloid *in vivo* model.

**Table S2.** The primers for the construction of the overexpression vectors

| Gene | Accession No. | ORF Length | Antibiotic | ORF Sequence Information |
| --- | --- | --- | --- | --- |
| Homo sapiens IFN-γ | NM_000619.2 | 501 bp | Ampicillin | 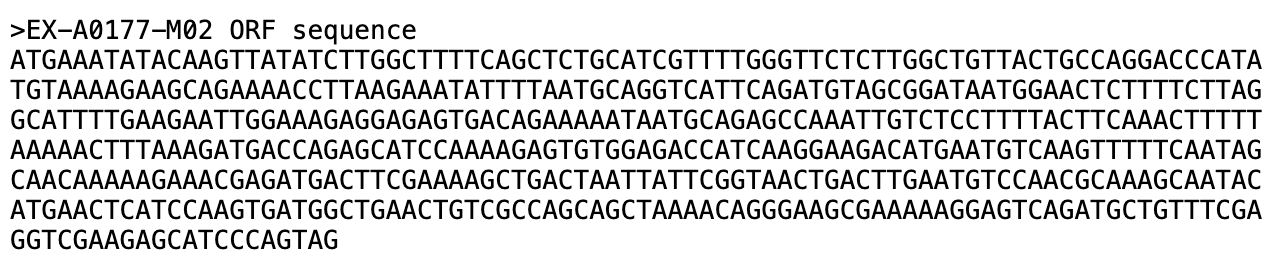 |
| Homo sapiens SERPINE2 | NM_006216.3 | 1197 bp | Ampicillin | 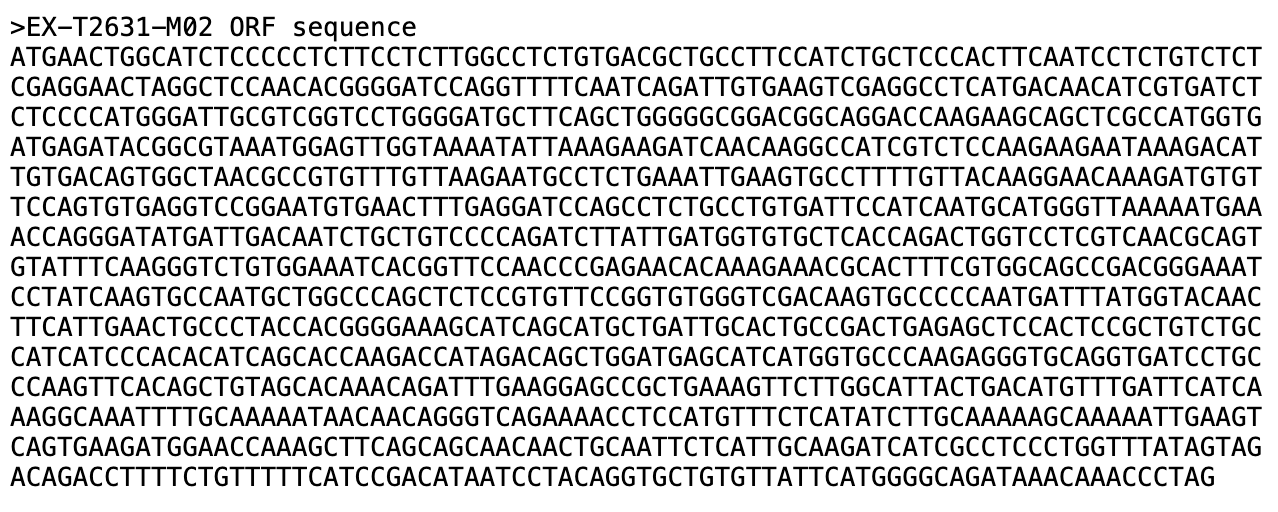 |

**Table S3.** Primers used in quantitative PCR analysis

| **Gene Name** | **Primer Sequence (5’ -3’)** |
| --- | --- |
| COL1A1 | Sense: GGCGGCCAGGGCTCCGACCC |
|  | Antisense: AATTCCTGGTCTGGGGCACC |
| COL3A1 | Sense: TGGTGTTGGAGCCGCTGCCA |
|  | Antisense: CTCAGCACTAGAATCTGTCC |
| α-SMA | Sense: CCCAGACATCAGGGAGTAATGG |
|  | Antisense: TCTATCGGATACTTCAGCGTCA |
| GPX4 | Sense: GAGGCAAGACCGAAGTAAACTAC |
|  | Antisense: CCGAACTGGTTACACGGGAA |
| SLC7A11 | Sense: TCTCCAAAGGAGGTTACCTGC |
|  | Antisense: AGACTCCCCTCAGTAAAGTGAC |
| SLC3A2 | Sense: GGGACTAACTCCTCCGACC |
|  | Antisense: TAGGAGAAGAGTCCAGGCCC |
| IFN-γ | Sense: TGCAGGTCATTCAGATGTAGC |
|  | Antisense: GGACATTCAAGTCAGTTACCG |
| GAPDH | Sense: TCACCATCTTCCAGGAGCG |
|  | Antisense: CTGCTTCACCACCTTCTTGA |
| MMP13 | Sense: TTCTCGGAGCCTCTCAGTCA |
|  | Antisense: CAGTTTGCAGAGCGCTACCT |
| SERPINE2 | Sense: GGCGTAAATGGAGTTGGTAA |
|  | Antisense: АССТСАСАСТGGАAСAСАTCTT |
| ACSL4 | Sense: CTGAAAGACTGGCAGGAAGG |
|  | Antisense: AATATCGCCAGTGCAAAACC |

**Table S4.** Antibodies used in western blot and IHC analyses

| Target protein | Brand | Host | Cat No. | Molecular Mass | Dilution |
| --- | --- | --- | --- | --- | --- |
| COL1A1 | Servicebio | Rabbit | GB11022 | 138/120-140KDa | WB: 1:500 |
| COL3A1 | Abcam | Rabbit | Ab184993 | 150/139KDa | WB: 1:1000 |
| α-SMA | Abcam | Rabbit | Ab124964 | 42KDa | WB: 1:1000 |
| GPX4 | Proteintech | Mouse | 67763-1-Ig | 20-23KDa | WB: 1:1000  IHC: 1:2000 |
| SLC7A11 | Proteintech | Rabbit | 26864-1-AP | 55,35-40KDa | WB: 1:1000  IHC: 1:200 |
| SLC3A2 | Proteintech | Rabbit | 15193-1-AP | 85-94/68KDa | WB: 1:5000  IHC: 1:200 |
| GAPDH | Proteintech | Rabbit | 10494-1-AP | 36KDa | WB: 1:5000 |
| MMP13 | Proteintech | Rabbit | 18165-1-AP | 70-65KDa | WB: 1:1000 |
| SERPINE2 | Proteintech | Rabbit | 11303-1-AP | 44-50KDa | WB: 1:500  IHC: 1:100 |
| ACSL4 | Proteintech | Rabbit | 22401-1-AP | 79,74KDa | WB: 1:2000  IHC: 1:200 |
| β-actin | Proteintech | Mouse | 66009-1-Ig | 42KDa | WB: 1:2000 |
| HRP-conjugated Goat Anti-Rabbit IgG(H+L) | Proteintech | Goat | SA00001-2 | —— | WB: 1:5000 |
| HRP-conjugated Goat Anti-Mouse IgG(H+L) | Proteintech | Goat | SA00001-1 | —— | WB: 1:5000 |

**Note:** The protein standards used in the experiment (BIO-RAD, Catalog #161-0394)
