# Supplementary material for: IFN-γ could induce ferroptosis in keloid fibroblasts by inhibiting the expression of serpine2
Source: Cell Death Discov. 2025 May 5;11:217. doi: 10.1038/s41420-025-02401-3 (PMC12053758; doi:10.1038/s41420-025-02401-3)
Supplement: Supplementary file 2 — SUPPLEMENTARY FIGURES [file 41420_2025_2401_MOESM2_ESM.pdf]

SUPPLEMENTARY FIGURES

IFN-γ could induce ferroptosis in keloid fibroblasts through inhibiting the expression of serpine2

Jingyan Huang[1*], MD, Yu Shun[2*], MD, Jing Luo[1], MD, Xusong Luo[1], PhD, Jun Yang[1#], PhD, MD,

Xiuxia Wang[1#], PhD

Fig. S1

a

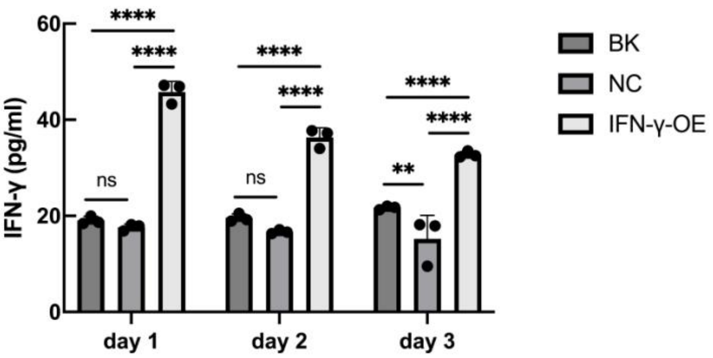

Fig. S2

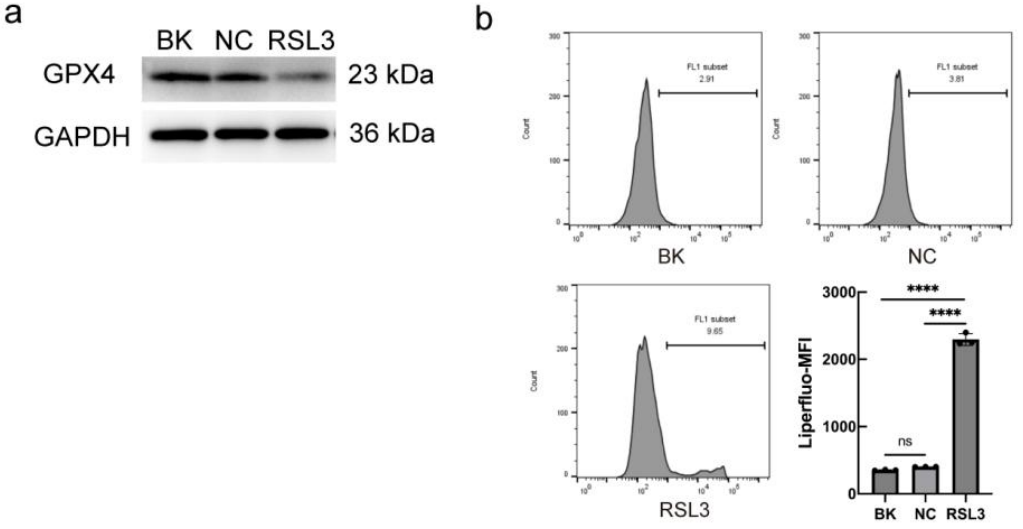
a
BK
NC
RSL3
GPX4
23 kDa
GAPDH
36 kDa
b
FL1 subset
2.91
FL1 subset
3.81
FL1 subset
9.65
Count
BK
NC
RSL3
Liperfluo-MFI
****
****
ns
BK
NC
RSL3

Fig. S3

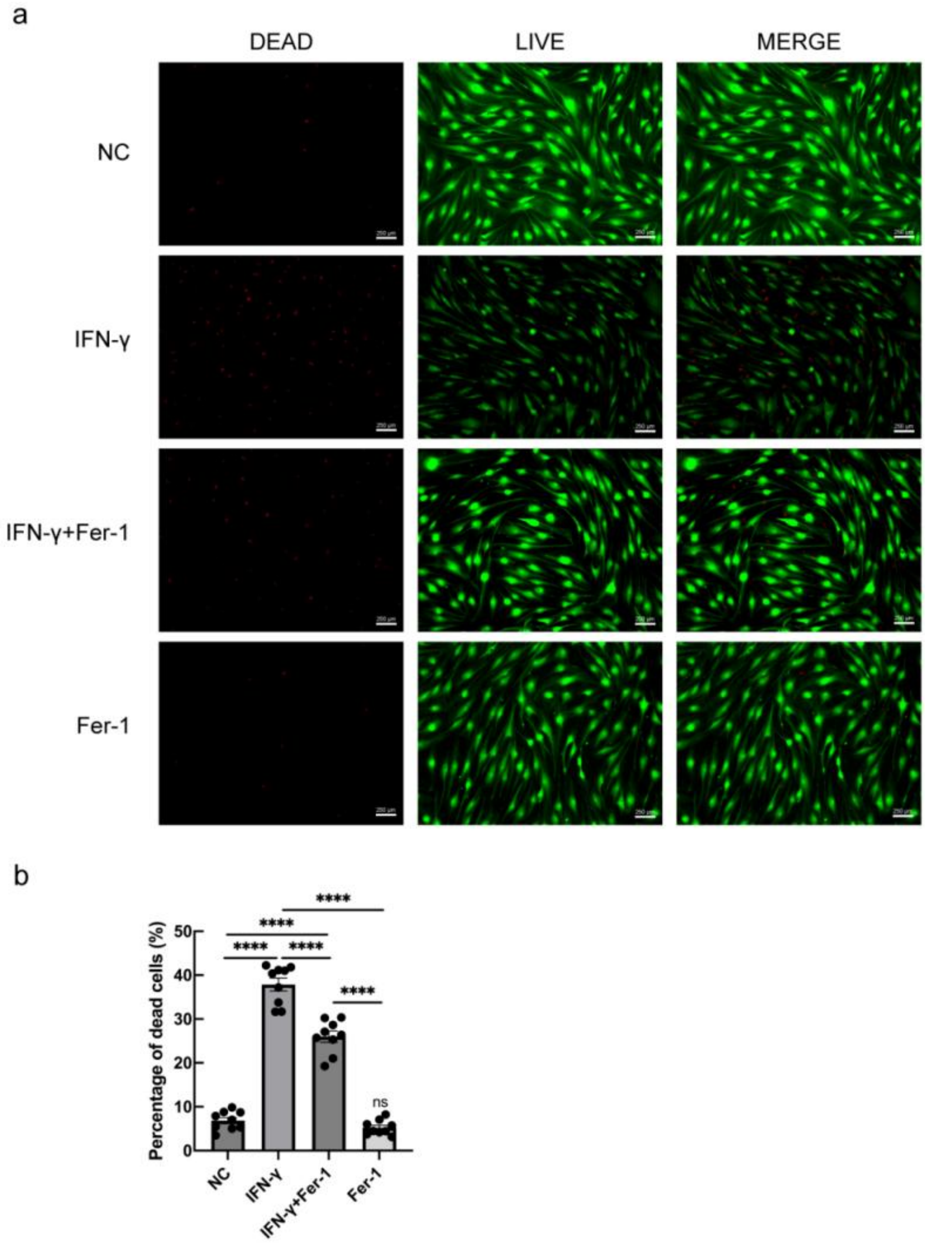

a
DEAD
LIVE
MERGE
NC
IFN-γ
IFN-γ+Fer-1
Fer-1
b
Percentage of dead cells (%)
50
40
30
20
10
0
****
****
****
****
****
ns
NC
IFN-γ
IFN-γ+Fer-1
Fer-1

Fig. S4

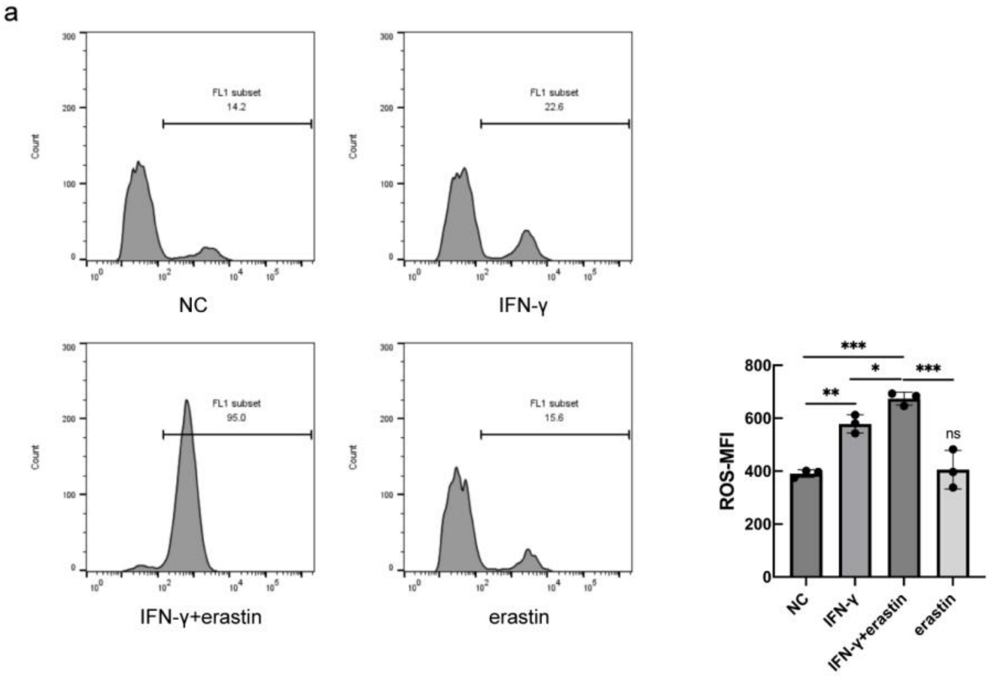
a
FL1 subset
14.2
NC
FL1 subset
22.6
IFN-γ
FL1 subset
95.0
IFN-γ+erastin
FL1 subset
15.6
erastin
Count
ROS-MFI
800
600
400
200
0
***
**
*
***
ns
NC
IFN-γ
IFN-γ+erastin
erastin

Fig. S5

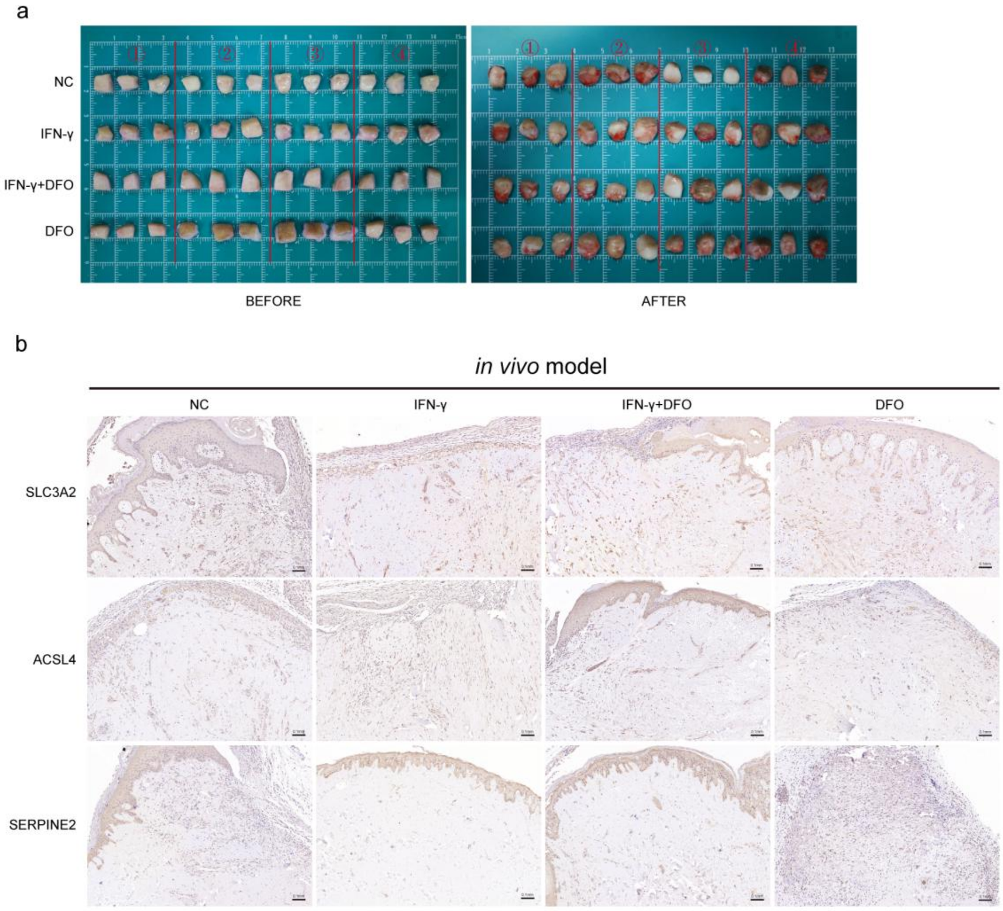
a
NC
IFN-γ
IFN-γ+DFO
DFO
BEFORE
AFTER
b
in vivo model
NC
IFN-γ
IFN-γ+DFO
DFO
SLC3A2
ACSL4
SERPINE2
