# Supplementary material for: IFN-γ could induce ferroptosis in keloid fibroblasts by inhibiting the expression of serpine2
Source: Cell Death Discov. 2025 May 5;11:217. doi: 10.1038/s41420-025-02401-3 (PMC12053758; doi:10.1038/s41420-025-02401-3)
Supplement: Supplementary file 3 — SUPPLEMENTARY FIGURE LEGENDS [file 41420_2025_2401_MOESM3_ESM.docx]

**Supplementary Figure legends**

**Fig. S1** ELISA kit to detect IFN-γ secreted by KFs after transfection with overexpression plasmids. (a) Cell supernatants were collected on days 1, 2, and 3 after transfection, and the concentration of IFN-γ secreted by the cells contained in each group was detected using an ELISA kit. (n=3) (ns: no significance, **P < 0.01, ****P < 0.0001.)

**Fig. S2** RSL3-treated (100nM) KFs were able to inhibit GPX4 protein expression, while lipid peroxidation levels were significantly increased in the RSL3-treated group compared to the blank and negative control groups (which contained only an equal amount of DMSO in the medium), suggesting that a promotion of ferroptosis process occurred. (a) Western blot experiments were performed to detect GPX4 protein expression levels in each group. (b) Liperfluo assay kit-treated cells were analyzed by flow cytometry, and positive cells were defined as FL1 subpopulations and counted as a percentage of total cells. By quantifying these fluorescent signals, the mean fluorescence intensity (MFI) was calculated, thus assessing and comparing the levels of lipid peroxide in each group of cells. (n=3) (ns: no significance, ****P < 0.0001.)

**Fig. S3** Compared with the NC group, the proportion of dead cells to total cells was significantly higher in the IFN-γ group. And the proportion of dead cells in the IFN-γ+Fer-1 group was significantly lower than that in the IFN-γ group, suggesting that Fer-1 as an inhibitor of ferroptosis could partially inhibit IFN-γ-induced cell death, while the effect of Fer-1 on the cells was not significantly different from that of the NC group.The protective control experiment of Fer-1 illustrated that IFN-γ was capable of inducing ferroptosis in KFs. (a) Live and dead cells double staining kit after staining with inverted light microscope to observe each group of cells, red fluorescence for dead cells, green fluorescence for live cells. (b) The percentage of dead cells to the total number of cells in each group was calculated and counted. (n=3) (ns: no significance, ****P < 0.0001.)

**Fig. S4** The level content of reactive oxygen species was significantly higher after IFN-γ and erastin acted together on KFs than IFN-γ and erastin alone. (a) Reactive Oxygen Assay Kit was analysed by flow cytometry after treatment of each group of cells, and the intracellular level of ROS was assessed by quantifying the fluorescence signals of the FL1 subpopulation and calculating the MFI. (n=3) (ns: no significance, *P < 0.05, **P < 0.01, ***P < 0.001.)

**Fig. S5** Keloid tissue samples obtained from the clinic (from each of the 4 patients, divided into 12 blocks of the same size, with 3 replicates in each group) were transplanted under the skin of nude mice, and the size of the blocks was measured before and after the transplantation using a ruler plate.The samples were collected after 2 weeks, and it was observed that all the tissue blocks retained a good activity with no contamination or necrosis. Immunohistochemical staining was performed after paraffin sections were made from the tissue blocks, and the results showed that, in agreement with the results of the previous study, the number of SLC3A2 and serpine2 positive cells was significantly reduced in the IFN-γ group compared with the NC group, while the number of ACSL4 positive cells was significantly increased. In addition, both IFN-γ and ferroptosis inhibitor DFO partially reversed the effect of IFN-γ after simultaneous injection, and no significant difference was seen with DFO alone compared with the NC group. In conclusion, IFN-γ was able to induce the onset of ferroptosis in keloid scars in an in vivo animal model. (a) Measurements of specimens before and after transplantation of keloid tissue blocks. (b) IHC staining of the SLC3A2, ACSL4, and SERPINE2 proteins in in vivo keloid models overexpressing IFN-γ, IFN-γ+DFO and DFO. (scale bar = 0.1 mm)
