# Supplementary figures and images for: IFN-γ could induce ferroptosis in keloid fibroblasts by inhibiting the expression of serpine2
Source: Cell Death Discov. 2025 May 5;11:217. doi: 10.1038/s41420-025-02401-3 (PMC12053758; doi:10.1038/s41420-025-02401-3)

Uncropped western blotting analysis

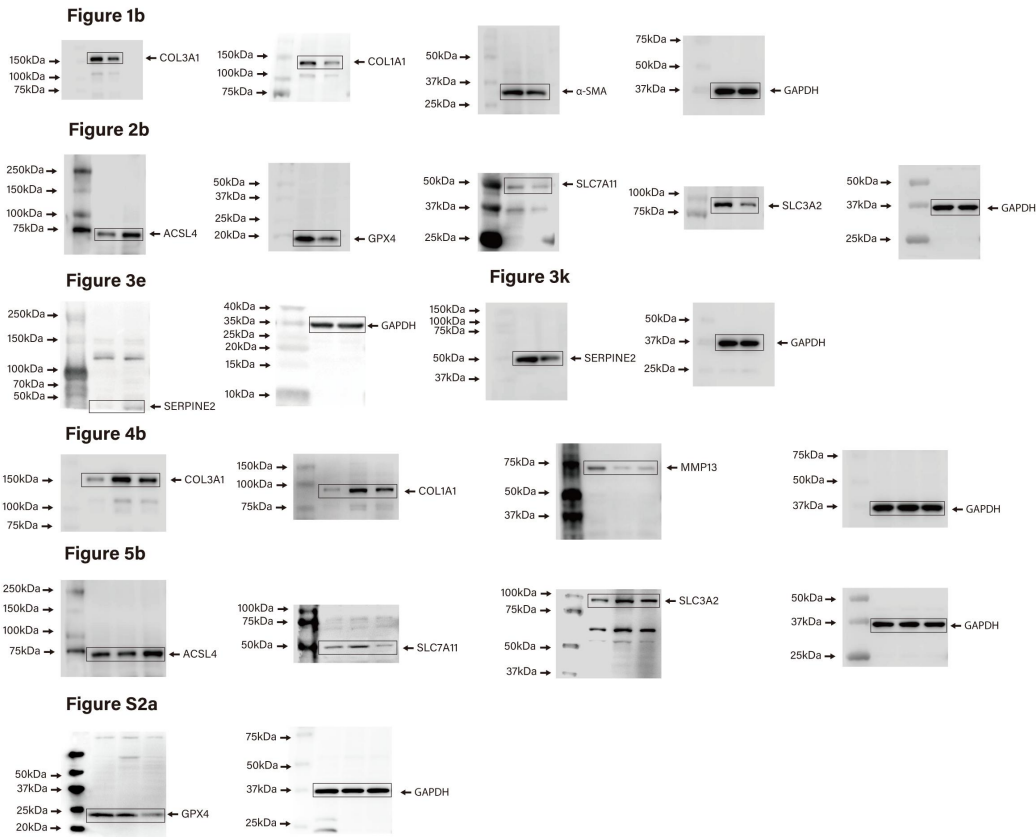

Supplement: Supplementary file 4 — Western Blot Original Data [file 41420_2025_2401_MOESM4_ESM.pdf]
